# Habitat radiomics assists radiologists in accurately diagnosing lymph node metastasis of adenocarcinoma of the esophagogastric junction

**DOI:** 10.1186/s13244-025-01969-9

**Published:** 2025-04-24

**Authors:** Pingfan Jia, Yueying Li, Haonan Li, Yuan Li, Huijuan Qin, Anyu Xie, Yuru Li, Luyao Wang, Luqin Ke, Huijie Feng, Hongwei Yu, Juan Li, Ning Yuan, Xing Guo

**Affiliations:** 1 Department of Radiology, Heping Hospital Affiliated to Changzhi Medical College, Changzhi, China; 2 Department of Radiology, China-Japan Friendship Hospital, Beijing, China; 3 Department of Radiology, Heji Hospital Affiliated to Changzhi Medical College, Changzhi, China

**Keywords:** Habitat radiomics, Adenocarcinoma of the esophagogastric junction, Lymph node, Node-RADS, Computed tomography

## Abstract

**Objectives:**

This study aimed to develop a habitat radiomics (HR) model capable of preoperatively predicting lymph node metastasis (LNM) in adenocarcinoma of the esophagogastric junction (AEG) and to implement its use in clinical practice.

**Methods:**

In this retrospective analysis, 337 patients from three centers were enrolled and divided into three cohorts: training, validation, and test (208, 52, and 77 patients, respectively). We constructed HR models, conventional radiomics models, and combined models to identify LNM in AEG. The area under the curve (AUC) was employed to identify the optimal model, which was then evaluated for assisting radiologists in the empirical and RADS groups in diagnosing LNM. Finally, the prediction process of the optimal model was visualized using SHAP plots.

**Results:**

The HR model demonstrated superior performance, achieving the highest AUC values of 0.876, 0.869, and 0.795 in the training, validation, and test cohorts, respectively. Regardless of seniority, the empirical group of radiologists showed a significant improvement in the AUC and accuracy when using the HR model, compared to working alone (*p* < 0.05). Furthermore, the RADS group radiologists exhibited strong reclassification ability, effectively reevaluating patients with false-negative LN initially classified as Node-RADS score 1 or 2 by themselves.

**Conclusion:**

The HR model facilitates the accurate prediction of LNM in AEG and holds potential as a valuable tool to augment radiologists’ diagnostic capabilities in daily clinical practice.

**Critical relevance statement:**

The habitat radiomics model could accurately predict the lymph node status of adenocarcinoma in the esophagogastric junction and assist radiologists in improving diagnostic efficacy, which lays the foundation for accurate staging and effective treatment.

**Key Points:**

- Accurate lymph node diagnosis in esophagogastric junction adenocarcinoma is beneficial for prognosis.
- Habitat radiomics model accurately predicted and assisted physicians in diagnosing lymph nodes.
- The habitat model effectively reclassified false-negative lymph nodes at Node-RADS 1 and 2.

**Graphical Abstract:**

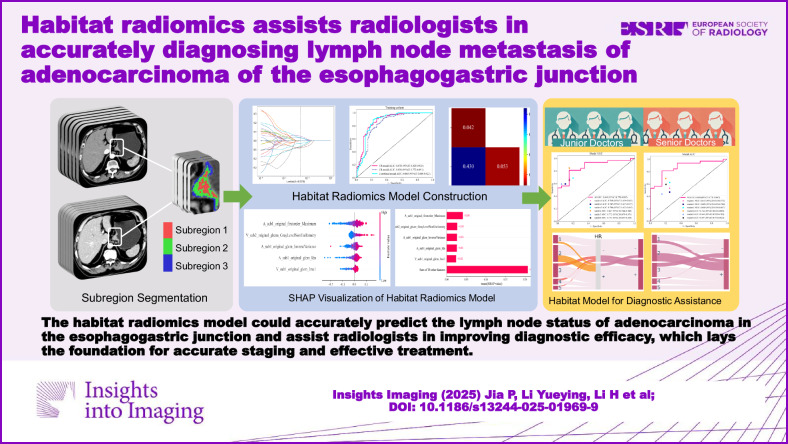

## Introduction

With the incidence and mortality of distal gastric cancer decreasing [1], the incidence of adenocarcinoma of the esophagogastric junction (AEG) has been rising, particularly in East Asia and some Western countries [2, 3]. The prognosis for AEG is generally worse than that for distal gastric cancer [4]. Lymph node metastasis (LNM) is an independent risk factor for poor prognosis in AEG [4, 5], making accurate preoperative assessment of lymph node (LN) status crucial for clinic staging and therapy [6, 7]. Due to the propensity of AEG to metastasize to both thoracic and abdominal LN [8], endoscopic ultrasound (EUS) has a high sensitivity in diagnosing LNM in AEG, but it also has a false-positive rate of up to 40%. PET-CT, while highly specific, is less sensitive than echoendoscopy (73% vs. 50%, *p* < 0.01), and CT, which is routinely used in AEG, has a low sensitivity [9, 10].

Our team had recently reviewed advances in radiomics for esophagogastric junction cancer (including depth of infiltration, HER-2 status, etc) [11]. However, previous studies used a generalized region-of-interest labeling method (entire tumor volume or largest dimension), which implicitly assumes that tumor heterogeneity is confounded [12]. However, spatial heterogeneity exists within tumors [13, 14]. Images, serving as macroscopic and quantitative tools for capturing tumor biology, have spurred the development of habitat imaging (HI). HI clusters tumor cells with similar characteristics into voxel groups within multimodal imaging, with applications in CT, MRI, and PET-CT [15, 16]. Furthermore, habitat radiomics (HR) offers a more accurate representation of intra-tumoral heterogeneity (ITH) by dividing tumor images into subregions and extracting radiomics features [17, 18]. HR has demonstrated superior predictive efficacy in drug resistance in ovarian cystic adenocarcinoma [19], genetic mutations in brain metastases of lung cancer [20], and survival duration after radiotherapy in esophageal cancer [17].

Currently, there is a gap in the mechanism of association between ITH and LNM in AEG. However, for esophageal squamous carcinoma, Teng H et al [21] found that LNM was associated with high heterogeneity of intra-tumoral DNA methylation. Lee HH et al [22] discovered that LNM in gastric cancer is linked to specific sub-clonal regions of the tumor primary. Based on this, we hypothesize that LNM in AEG is influenced by spatial heterogeneity within the tumor and that different tumor subregions may differentially contribute to LNM. Since previous studies relied on invasive and difficult-to-repeat pathological tissue analysis, our study aims to accurately predict the LN status of AEG using the HR model based on preoperative CT and to implement its use in clinical practice.

## Materials and methods

### Patients

**Fig. 1:**
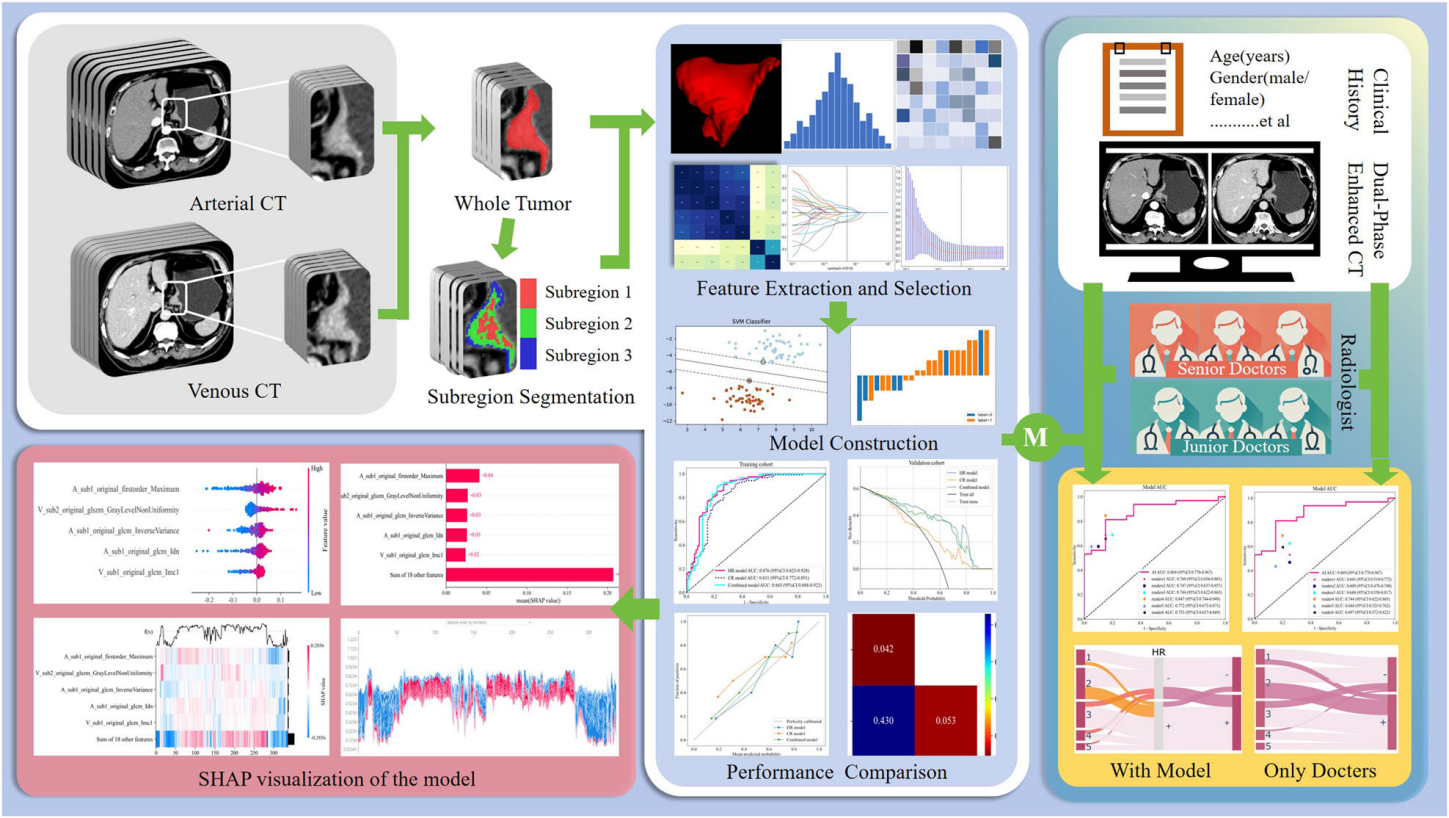
Flowchart of the research. M, model

This retrospective study, approved by the institutional review boards (IRB number: 2022002) of Heping Hospital, Heji Hospital, and China-Japan Friendship Hospital with exempted informed consent, consecutively included 468 patients with AEG from Heping Hospital (June 2021 to August 2023), 115 patients from Heji Hospital (July 2022 to July 2023), and 83 patients from China-Japan Friendship Hospital (July 2021 to October 2022). The study followed the METRICS guidelines. Inclusion criteria were AEG confirmed by surgical pathology with clear staging and scope of LN dissection, and preoperative abdominal CT (arterial and venous phases) within 2 weeks of surgery. Exclusion criteria were preoperative therapy, history of other tumors, poor image quality, and small lesions preventing image registration and tumor labeling. LNM positivity was defined as one or more LNM based on surgical pathology. Patients from Heping Hospital were randomly split into training and validation cohorts (4:1 ratio), while patients from the other institutions formed the test cohort. Figure 1 outlines the study workflow.

### CT image acquisition and preprocessing

Preoperative enhanced abdominal CT (arterial and venous phases) was performed on the patients, with CT equipment and iodine contrast parameters detailed in supplementary materials (Table S1) for the three centers. To enhance result reproducibility and comparability, CT images underwent preprocessing [23]. This involved registering arterial and venous-phase images using the General Registration (ANTs) in 3D Slicer software (version 5.0.3) to align tumor spatial voxels across phases, ensuring accurate tumor subregion delineation. Aligned images were then resampled to 1 mm × 1 mm × 5 mm using a B-spline interpolation algorithm, discretizing the grayscale range with a bin width of 25.

### Tumor volume of interest (VOI) and subregion delineation

An abdominal radiologist (6 years of experience [J.P.]) hand-segmented AEG primary lesions layer by layer on the aligned CT images of all cohorts in ITK-SNAP (version 3.8.0), blinded to the patient’s pathological information. Another physician (10 years of experience [Q.H.]) independently outlined 30 randomly selected cases under the same conditions to clarify whether the radiomics features were subjectively influenced by the physicians. The voxels within the tumor VOI on two-phasic CT images were clustered using the K-means clustering algorithm on Onekey AI (version 2.4.10) as feature vectors to delineate different subregions. The number of clusters, a user-set parameter, ranged from 2 to 10. The Calinski-Harabasz score determined the best clustering configuration, with the highest score achieved at 3 clusters (Fig. S2a and Table S2). Thus, the tumor was divided into three subregions: sub1, sub2, and sub3. Tumor VOI labeling and subregion delineation are shown in Fig. S3.

### Feature extraction and selection

The whole tumor volume (A_whole, V_whole) and its subregions (A_sub1, A_sub2, A_sub3, V_sub1, V_sub2, V_sub3) on arterial- and venous-phase CT images were used as a source of radiomics feature abstraction. Features included first-order statistics (18), shape features (14), and texture metrics (73). Considering the subregional segmentation, shape features were excluded for subregions, retaining only first-order and texture features. All of the above were compliant with the Image Biomarker Standardization Initiative (IBSI) standard [24]. Features from the whole tumor formed conventional radiomics (CR) features, while those from subregions constituted HR features. These features were then integrated into combined radiomics features. Z-score normalization scaled these features to a 0–1 range. The feature screening process is detailed in Part I of the supplementary material.

### Model construction and evaluation

The HR model was constructed using HR features, the CR model based on CR features, and the combined model using the combined features (HR + CR). To identify the optimal model, various algorithms were employed, including Support Vector Machine (SVM), Random Forest, Extremely Randomized Trees, eXtreme Gradient Boosting, Light Gradient Boosting Machine, and Logistic Regression [25, 26]. Fifty-fold cross-validation modulated the parameter configurations of each model. Youden’s index was used to determine the optimal cut-off value for models. DeLong’s test was performed to select the best model to assist the radiologist in making a diagnosis.

### Model assists radiologists in diagnosis

Six radiologists (three seniors with 6–15 years of gastrointestinal CT practice and three juniors with 1–5 years) independently screened CT images of the validation and test cohorts in randomized order across two rounds to evaluate the model’s utility in assisting diagnosis. In Round 1, radiologists diagnosed patients based solely on clinical data and dual-phase CT images, marking LNM positive if any LN was suspected of metastasis. In Round 2, after receiving the model’s dichotomous classification results (positive for LNM if predicted value > cut-off), radiologists repeated the assessments under model assistance. To ensure the radiologists’ diagnostic process was both clinically relevant and state-of-the-art, we randomly divided radiologists into two groups and used different methods to assess LN status. Radiologists in the empirical group (two seniors [Y.N., Q.H.] and two juniors [K.L., F.H.]) were diagnosed based on subjective assessments of LN size and morphology, while radiologists in the RADS group (two remaining doctors [L.J., L.Y.]) strictly followed the Node Reporting and Data System 1.0 (Node-RADS) [27], scoring the largest LN. An LN enlargement threshold of 10 mm. The Node-RADS score ≥ 3 indicated LNM [28]. All radiologists were unaware of each other’s assessments, original diagnostic reports, and final pathological results. Each round included three repeat assessments spaced one week apart in random order. If the results of the three assessments were inconsistent, the two consistent assessments would be the final result. Details on the RADS group’s learning process and Node-RADS scoring are provided in Part I of the supplementary materials.

### Models for identifying Node-RADS scoring errors by radiologists

The Sankey plot connects the independent diagnoses (Node-RADS score 1–5) of the radiologists in the RADS group to the LN status via flow curves. Additionally, we highlighted cases reclassified by the model with distinct colors for the physicians. This approach allows direct visualization of scoring errors in the independent diagnoses and assesses the model’s ability to recalibrate LN classifications across different Node-RADS categories. A line graph illustrates the differences in metrics between physician-independent diagnoses and model predictions at various Node-RADS scores. The Sankey plots were generated using the Plotly package (version 5.2.1) in Python.

### SHAP visual analysis

To better illustrate the contribution of different subregion features in the HR model to predicting LN status and their role in the model, we employed the Shapley Additive exPlanations (SHAP) algorithm. This algorithm allowed us to assess the importance of the HR features and determine whether their contribution to the model was positive or negative. SHAP visual display was conducted from the Explainer package (version 0.40.0).

### Statistical analysis

Python (version 3.7.12) was deployed for the evaluation it follows. Statistical analyses including chi-square tests, *t*-tests, and Mann–Whitney *U*-tests based on the type of data. The diagnostic performance of machine models and radiologists was assessed with the area under the curve (AUC), sensitivity, specificity, positive predictive value (PPV), and negative predictive value (NPV). Model performance was compared using DeLong tests. Decision curve analysis (DCA) assessed the net benefit of the models. Calibration curves and the Hosmer-Lemeshow test assessed calibration capability. DeLong tests and McNemar tests were used to analyze changes in AUC and accuracy, respectively, before and after radiologists incorporated model assistance. Fleiss’ kappa was calculated to assess reader internal consistency. A two-sided *p*-value less than 0.05 was deemed statistically significant.

## Results

### Baseline characteristics of patients

Of the 666 AEG patients from three centers, 187 who received preoperative treatment, 39 with a history of other tumors, 75 with poor image quality, and 28 with lesions too small to align and label were excluded. This resulted in 337 AEG patients, divided into: a training cohort of 208 patients, a validation cohort of 52 patients, and a test cohort of 77 patients (44 + 35) from two external centers. Positive LNM (pT1-3) was observed in 122 (58.65%) patients in the training cohort, 32 (61.54%) in the validation cohort, and 54 (70.13%) in the test cohort. Siewert type I patients were less frequent, occurring in 6 (2.88%), 0 (0%), and 4 (5.19%) patients across the training, validation, and test cohorts, respectively. Baseline demographic characteristics showed no significant differences between cohorts. Further details are provided in Table 1 and Fig. S1.

**Table 1:** Clinicopathological characteristics of three cohorts of patients

	Training (*n* = 208)	Validation (*n* = 52)	Test (*n* = 77)	*p-*value
Sex, *n* (%)				0.85
Male	169 (81.25)	44 (84.62)	63 (81.82)	
Female	39 (18.75)	8 (15.38)	14 (18.18)	
Age, mean (SD), year	65.39 (7.93)	65.37 (8.31)	67.62 (8.80)	0.11
Maximum thickness of lesion, mean (SD), mm	16.94 (5.80)	16.25 (6.11)	15.25 (6.36)	0.10
Siewert type, *n* (%)				0.10
I	6 (2.88)	0 (0)	4 (5.19)	
II	109 (52.40)	32 (61.54)	50 (64.94)	
III	93 (44.72)	20 (38.46)	23 (29.87)	
Degree of differentiation, *n* (%)				0.76
High	11 (6.73)	3 (5.77)	7 (9.09)	
Medium	102 (49.04)	23 (44.23)	37 (48.05)	
Low	95 (45.67)	26 (50.00)	33 (42.86)	
pT staging, *n* (%)				0.41
1	10 (4.81)	3 (5.77)	8 (10.39)	
2	15 (7.22)	4 (7.69)	10 (12.99)	
3	137 (65.87)	35 (67.31)	45 (58.44)	
4	46 (22.12)	10 (19.23)	14 (18.18)	
pN staging, *n* (%)				0.21
0	86 (41.35)	20 (38.46)	23 (29.87)	
1–3	122 (58.65)	32 (61.54)	54 (70.13)	
Vascular invasion, *n* (%)				0.11
Negative	110 (52.88)	32 (61.54)	33 (42.86)	
Positive	98 (47.12)	20 (38.46)	43 (55.84)	

*pT staging* pathological T-staging, *pN staging* pathological N-staging, *SD* standard deviation

### Tumor volume of interest (VOI) and subregion delineation

Figure S2b shows that among the CT values of the voxels in different subregions, subregion 1 is highly intensified in both the arterial and venous phases, followed by subregion 2 and subregion 3. In Fig. S3d, the subregions are arranged in concentric circles. Subregion 1 is primarily located in the middle layer of the tumor, subregion 2 is found in the superficial and partially deeper layers, and subregion 3 is located at the junction between the tumor and normal gastric tissue.

### Feature extraction and selection

546 HR features, 210 CR features, and 756 combined features of AEG lesions were extracted from dual-phase CT (Tables S3–5). Feature screening retained 23 HR features, 9 CR features, and 19 combined radiomics features (Fig. S4). Figure S5 illustrates differences in the distribution of HR features in the tumor subregion and the CT phase.

### Model construction and evaluation

**Fig. 2:**
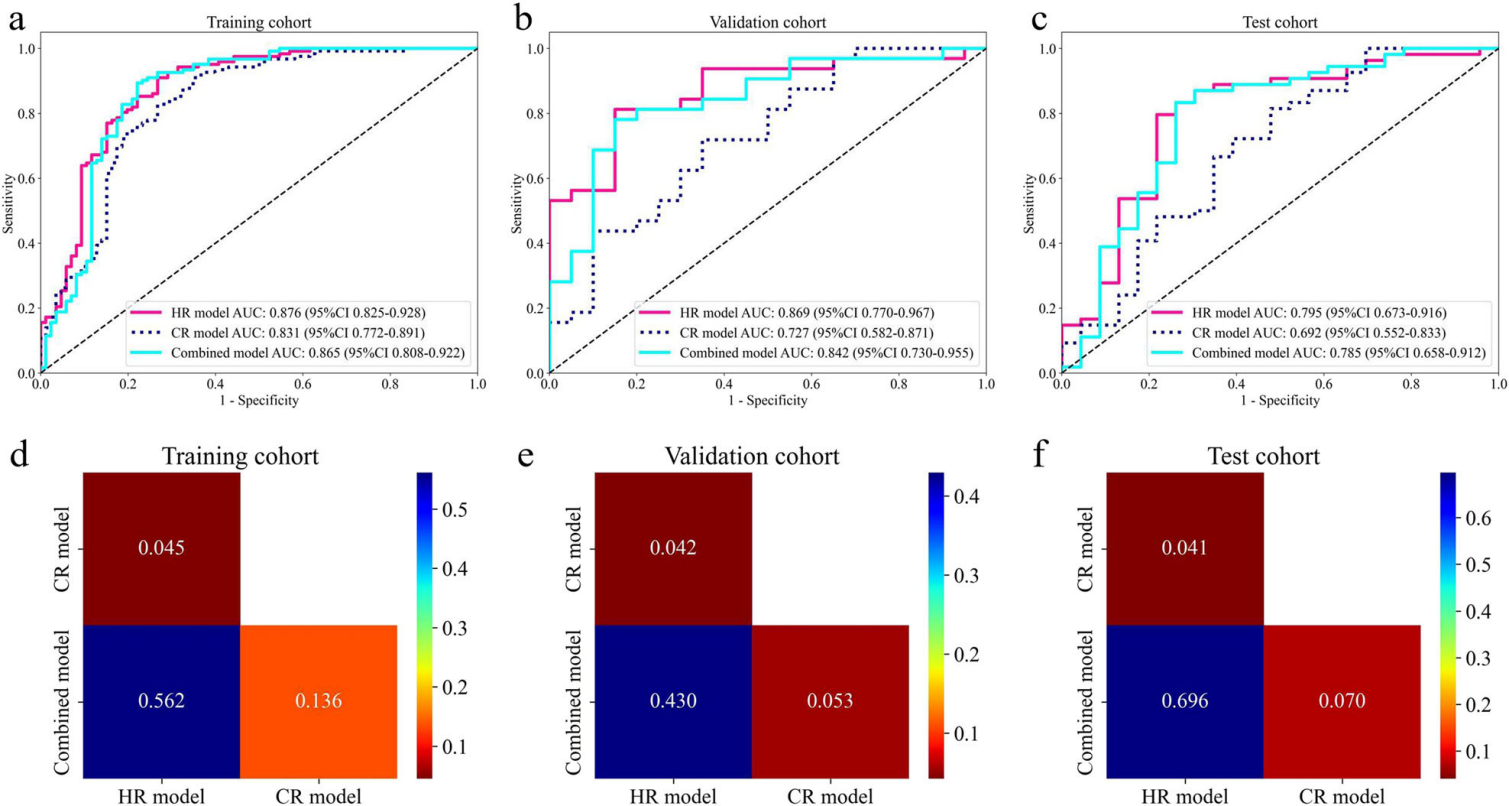
Model performance comparison. Legend: Receiver operating characteristic (ROC) curves and Delong test in the training cohort (**a**, **d**), validation cohort (**b**, **e**), and test cohort (**c**, **f**). HR, habitat radiomics; CR, conventional radiomics; AUC, the area under the curve

From the AUC values in Tables S6–S8, SVM is the best-performing algorithm among the remaining algorithms in the validation and test sets of all models, except for the LR algorithm, which shows marked overfitting in the CR model. Therefore, SVM was selected to construct the HR model, CR model, and combined model. The predictive performance of SVM is detailed in Table 2. As presented in Fig. 2, the HR model has the highest AUC, with statistically significant differences compared to the CR model (0.876 vs. 0.831, 0.869 vs. 0.727, and 0.795 vs. 0.692, *p* < 0.05). The HR model has the greatest net clinical benefit (Fig. S6) and area under the precision-recall curve (Fig. S7). Calibration curves (Fig. S8) and Hosmer-Lemeshow tests (Table S9) show a good fit for all models (*p* > 0.05). Consequently, the HR model was adopted as a validation tool to assist radiologists in diagnosis.

**Table 2:** Performance comparison of different models

Model	AUC (95% CI)	Accuracy	Sensitivity	Specificity	PPV	NPV	Precision	Recall	F1	Cohort
HR	0.876 (0.825–0.928)	0.832	0.902	0.733	0.827	0.840	0.827	0.902	0.863	Training
CR	0.831 (0.772–0.891)	0.798	0.902	0.651	0.786	0.824	0.786	0.902	0.840	Training
Combined	0.865 (0.808–0.922)	0.841	0.885	0.779	0.850	0.827	0.850	0.885	0.867	Training
HR	0.869 (0.770–0.967)	0.808	0.781	0.850	0.893	0.708	0.893	0.781	0.833	Validation
CR	0.727 (0.582–0.871)	0.673	0.687	0.650	0.759	0.565	0.759	0.687	0.721	Validation
Combined	0.842 (0.730–0.955)	0.788	0.750	0.850	0.889	0.680	0.889	0.750	0.814	Validation
HR	0.795 (0.673–0.916)	0.779	0.778	0.783	0.894	0.600	0.894	0.778	0.832	Test
CR	0.692 (0.552–0.833)	0.714	0.796	0.522	0.796	0.522	0.796	0.796	0.796	Test
Combined	0.785 (0.658–0.912)	0.792	0.815	0.739	0.880	0.630	0.880	0.815	0.846	Test

*HR* habitat radiomics, *CR* conventional radiomics, *AUC* area under the curve, *CI* confidence interval, *PPV* positive predictive value, *NPV* negative predictive value

### Model assists radiologists in diagnosis

**Fig. 3:**
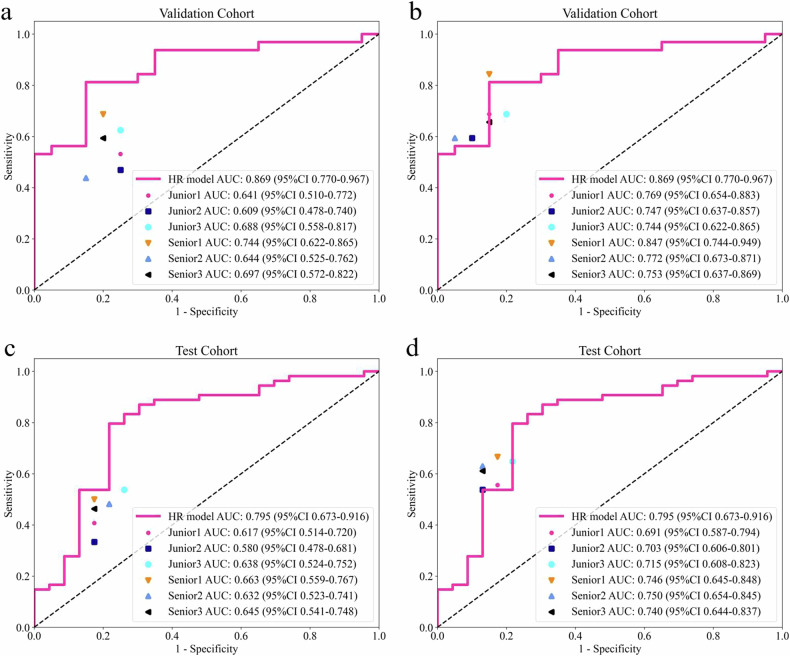
Comparisons of diagnostic performance between habitat radiomics (HR) models and radiologists. Legend: The figure shows the variation in radiologists before and after using the HR model to aid diagnosis of lymphatic node metastasis (LNM) in the validation cohort (**a**, **b**) and the test cohort (**c**, **d**)

The results of LNM diagnosis by radiologists before and after HR model assistance are shown in Tables 3–5 and Fig. 3. Intra-physician diagnostic consistency was fine (Table S10). Before HR model assistance, in both groups, six radiologists of varying seniority had lower AUC and accuracy in independent diagnoses compared to the HR model. Five radiologists (excluding Senior 1) showed significant differences in AUC compared to the HR model (*p* < 0.05). Post-assistance, all radiologists saw improvements in AUC, accuracy, and sensitivity from their initial diagnoses. Notably, the empirical group radiologists demonstrated statistically significant differences in AUC and accuracy before and after HR model assistance (*p* < 0.05). Moreover, there were no statistically significant differences in AUC or accuracy post-assistance compared to the HR model itself (except for Junior 2 in the test cohort). All physicians maintained or enhanced their diagnostic specificity after HR model assistance.

**Table 3:** Comparison of radiologists with and without HM model assistance in the validation cohort

	Group	Doctors	AUC (95% CI)		*p*-value^a^			Accuracy		*p*-value^b^		
			Without HM	With HM	Without HM vs. HM	With HM vs. HM	Without HM vs. With HM	Without HM	With HM	Without HM vs. HM	With HM vs. HM	Without HM vs. With HM
Junior	Empiric	Junior 1	0.641 (0.510–0.772)	0.769 (0.654–0.883)	0.003	0.123	0.007	0.615	0.750	0.041	0.581	0.016
		Junior 2	0.609 (0.478–0.741)	0.747 (0.637–0.857)	0.002	0.096	0.007	0.577	0.712	0.017	0.302	0.016
	RADS	Junior 3	0.688 (0.559–0.817)	0.744 (0.622–0.865)	0.015	0.067	0.090	0.673	0.731	0.119	0.388	0.250
Senior	Empiric	Senior 1	0.744 (0.622–0.865)	0.847 (0.744–0.950)	0.037	0.622	0.012	0.731	0.846	0.424	0.727	0.031
		Senior 2	0.644 (0.525–0.762)	0.772 (0.673–0.871)	0.004	0.153	0.007	0.596	0.731	0.019	0.388	0.016
	RADS	Senior 3	0.697 (0.572–0.822)	0.753 (0.637–0.869)	0.013	0.062	0.090	0.673	0.731	0.092	0.344	0.250

*HR* habitat radiomics, *CI* confidence interval, *Without HM* physician-independent diagnosis, *With HM* HR modeling aids in the diagnosis

^a^ Delong test

^b^ McNemar test

**Table 4:** Comparison of radiologists with and without HM model assistance in the test cohort

	Group	Doctors	AUC (95% CI)		*p*-value^a^			Accuracy		*p*-value^b^		
			Without HM	With HM	Without HM vs. HM	With HM vs. HM	Without HM vs. With HM	Without HM	With HM	Without HM vs. HM	With HM vs. HM	Without HM vs. With HM
Junior	Empiric	Junior 1	0.617 (0.514–0.720)	0.691 (0.587–0.795)	0.009	0.115	0.002	0.533	0.636	0.003	0.061	0.008
		Junior 2	0.580 (0.478–0.681)	0.703 (0.606–0.801)	< 0.001	0.075	0.001	0.481	0.636	< 0.001	0.013	0.002
	RADS	Junior 3	0.638 (0.524–0.752)	0.715 (0.608–0.823)	0.022	0.191	0.012	0.597	0.688	0.016	0.210	0.016
Senior	Empiric	Senior 1	0.663 (0.559–0.767)	0.746 (0.645–0.848)	0.050	0.391	0.040	0.597	0.714	0.020	0.383	0.012
		Senior 2	0.632 (0.523–0.741)	0.750 (0.654–0.845)	0.014	0.450	0.002	0.571	0.701	0.004	0.263	0.002
	RADS	Senior 3	0.645 (0.541–0.748)	0.740 (0.644–0.837)	0.017	0.314	0.003	0.571	0.688	0.004	0.167	0.004

*HR* habitat radiomics, *CI* confidence interval, *Without HM* physician-independent diagnosis, *With HM* HR modeling aids in the diagnosis

^a^ Delong test

^b^ McNemar test

**Table 5:** Differences in sensitivity and specificity between radiologists assisted with and without the HM model

Doctors	Validation cohort				Test cohort			
	Sensitivity		Specificity		Sensitivity		Specificity	
	Without HR	With HR	Without HR	With HR	Without HR	With HR	Without HR	With HR
Junior 1	0.531	0.687	0.750	0.850	0.407	0.556	0.826	0.826
Junior 2	0.469	0.594	0.750	0.900	0.333	0.537	0.826	0.870
Junior 3	0.625	0.687	0.750	0.800	0.537	0.648	0.739	0.783
Senior 1	0.687	0.844	0.800	0.850	0.500	0.667	0.826	0.826
Senior 2	0.437	0.594	0.850	0.950	0.481	0.630	0.783	0.870
Senior 3	0.594	0.656	0.800	0.850	0.463	0.611	0.826	0.870

*HR* habitat radiomics, *Without HM* physician-independent diagnosis, *With HM* HR modeling aids in the diagnosis

### Models for identifying Node-RADS scoring errors by radiologists

**Fig. 4:**
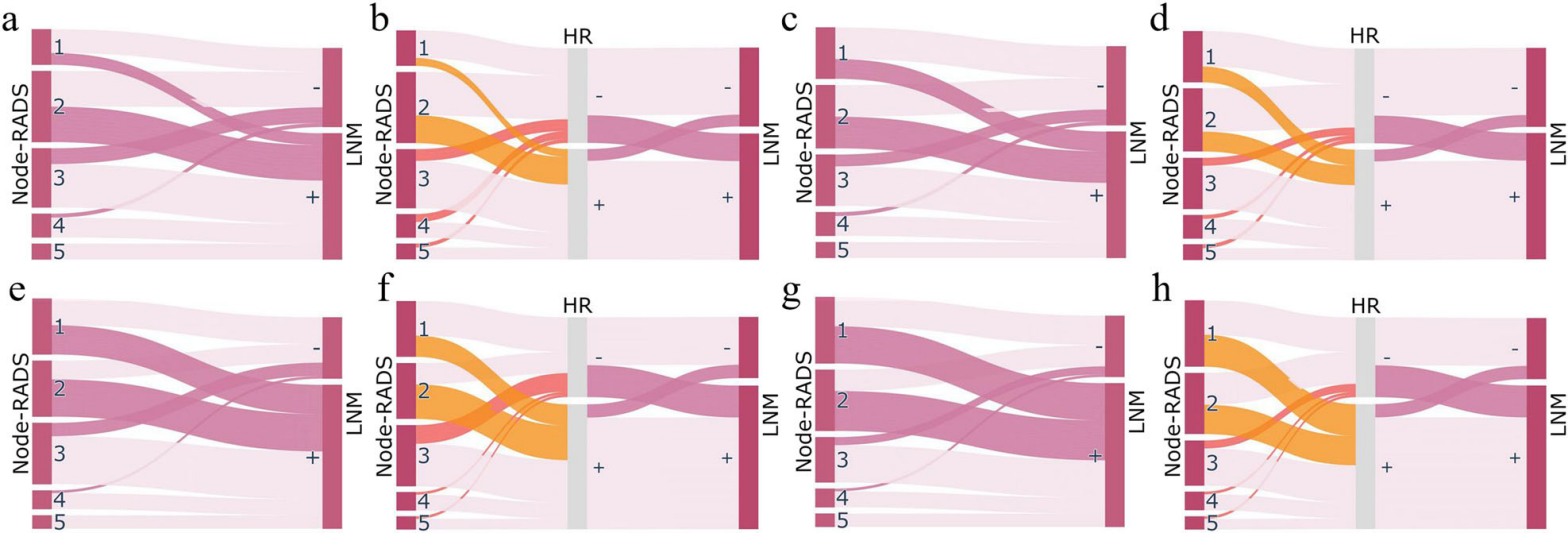
Sankey diagram for Node-RADS. Legend: **a**, **c**, **e**, **g** present Sankey plots of Node-RADS scores for LN assessed by RADS group physicians Junior 3 and Senior 3 in the validation and test cohorts. Lighter pink lines indicate correct diagnoses, while darker pink lines denote incorrect diagnoses. **b**, **d**, **f**, **h** are based on the independent physician scores mentioned earlier, incorporating the HR model as an intermediate node. Reclassified cases are represented by different yellow lines: earthy yellow denotes cases reclassified as positive in Node-RADS scores 1–2, while orange represents those reclassified as negative in scores 3–5. +, LNM positive; -, LNM negative

**Fig. 5:**
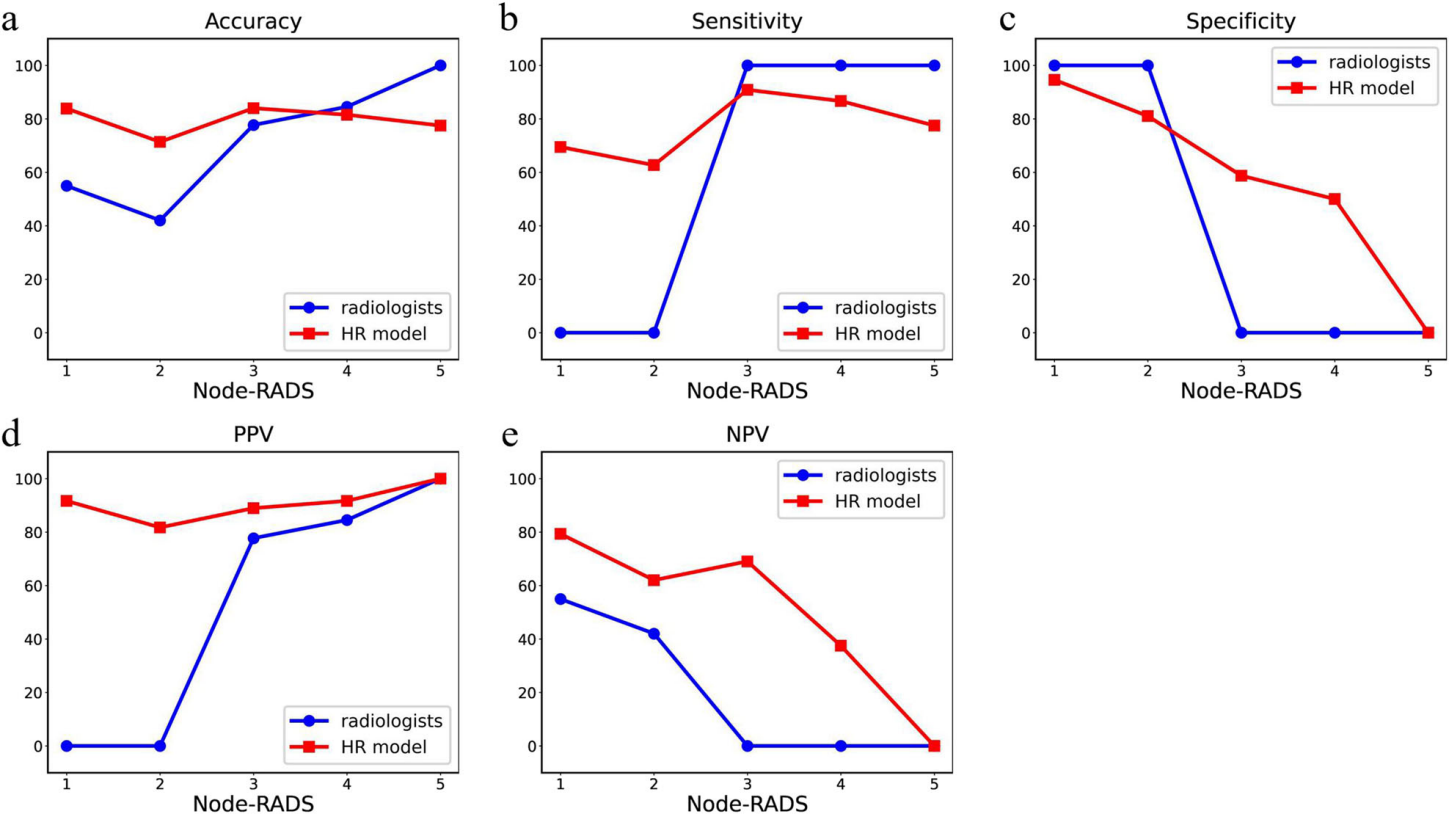
Line plot of diagnostic metrics at different scores for Node-RADS. **a** Accuracy. **b** Sensitivity. **c** Specificity. **d** PPV. **e** NPV

When physicians in the RADS group independently used Node-RADS to diagnose LN status in AEG, misclassification mainly occurred in RADS scores 1–3 (Fig. 4a, c, e, g). The HR model’s reclassification of Node-RADS was also concentrated in scores of 1–3 (Fig. 4b, d, f, h). Figure 5 and Table S11 show that physicians in the RADS group diagnosed LN of AEG as scores 1 or 2 on Node-RADS, having a lower diagnostic accuracy than scores 3–5. However, the HR model had similar diagnostic accuracy between Node-RADS scores 1–5 and substantially improved diagnostic accuracy, sensitivity, and PPV for Node-RADS scores 1 and 2.

### SHAP visual analysis

**Fig. 6:**
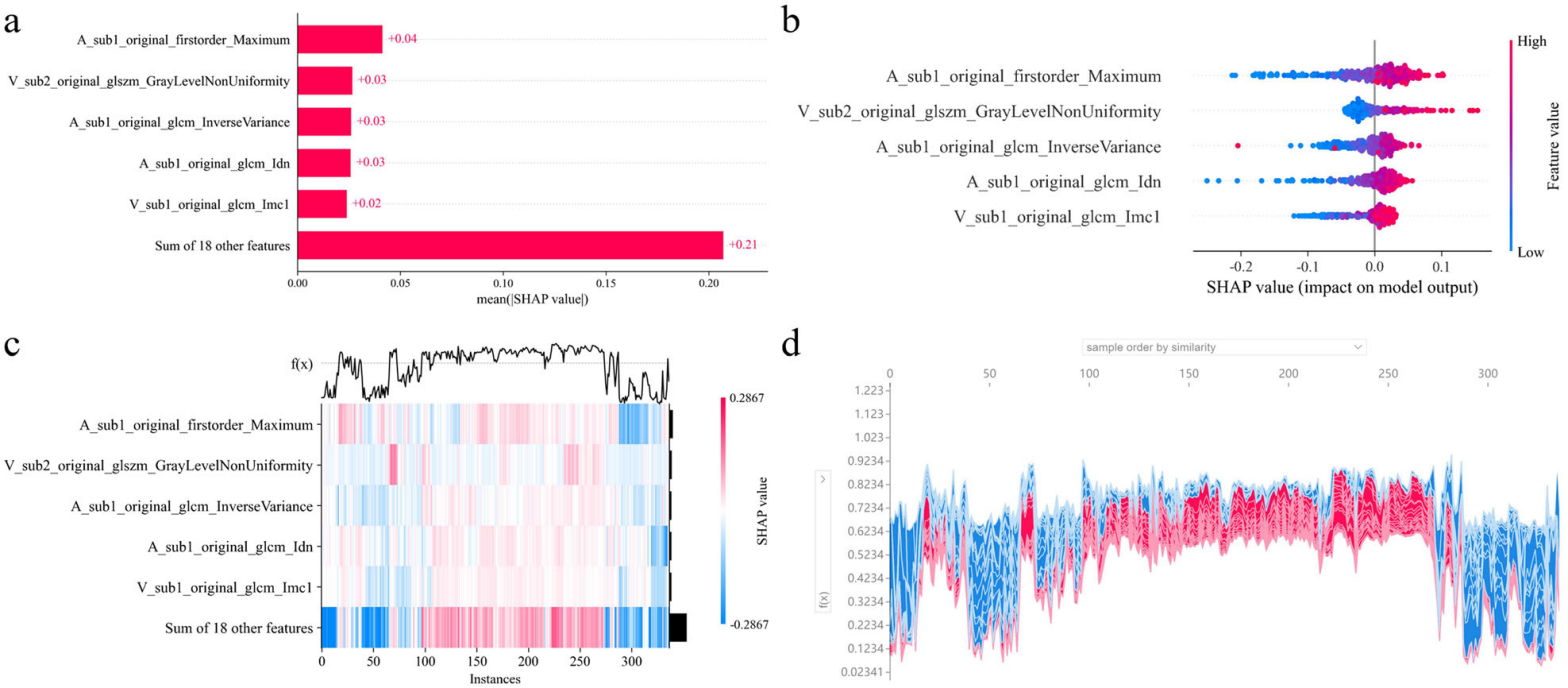
SHAP visualization of HR models. Legend: **a** SHAP bar chart. **b** SHAP summary plot. **c** SHAP heatmap. **d** SHAP stacked force plot. SHAP, Shapley additive explanation

We calculated overall and individual Shapley values for the SVM-based HR model. The SHAP bar chart (Fig. 6a) highlights the top five features of the model and their weights and subregional distributions. The SHAP summary plot (Fig. 6b) indicates that these top five features positively influenced LNM prediction in AEG. The SHAP heatmap (Fig. 6c) illustrates the direction and magnitude of the influence of these features on all AEG cases. Figure S9 provides a comprehensive view of all 23 features using SHAP bar charts, summary plots, and heat maps. The SHAP stacked force plot (Fig. 6d), created by rotating the force plot 90° and stacking features, exhibits the cumulative contribution of all 23 features in predicting LNM for each AEG patient. In individual case visualizations, Fig. S10a–d depict two representative cases where the HR model accurately predicted LN status and illustrated diagnostic changes made by six radiologists before and after using the HR model. The SHAP force plot (Fig. S10e) outlines the key feature categories that affect the Shapley values for individual AEG cases and their prediction of the orientation and degree of LNM.

## Discussion

This study established and validated the superiority of a dual-phase enhanced CT-based HR model over the CR model for preoperative LNM assessment in AEG. The HR model significantly enhanced the diagnostic performance of radiologists of varying seniority, particularly in recognizing false-negative LN (Node-RADS scores 1 and 2). Additionally, we visualized the feature weights within the HR model to elucidate its underlying role.

In the comparison of the three models, the AUC of the combined model was slightly lower than that of the HR model in all cohorts, possibly due to the significantly lower AUC of the CR model, which pulled down the performance of the combined model. The HR model contains 23 radiomics features, the majority of which have been validated for characterizing LNM in gastric cancer models [29, 30]. Notably, the feature with the highest weight in the SHAP bar chart was located in subregion 1, which is situated in the middle layer of tumor infiltration and corresponds to the highly intensified area of the AEG on the scatter plot. This suggests that the blood-rich area of the AEG is most strongly correlated with LN status. Notably, Naruke A et.al has found that vascular endothelial growth factor (VEGF) is expressed at a high level in the middle layer of the primary foci of gastric cancer [31]. VEGF’s role in promoting vessel proliferation and increased permeability underscores its notable association with LNM in AEG [32]. Thus, our findings align with Sundar R et al, who observed that genes related to angiogenesis and invasiveness were non-enriched in the superficial layers of tumors in gastric cancer with LNM [33]. Subregion 2 was compoundly distributed in the superficial and partially deep layers of the tumor tissue, indicating that the degree of enhancement in AEG and the depth of infiltration were not simply correlated. Subregion 3, located mainly in the tumor border area, exhibited a microenvironment transitioning between disordered and normal. The lack of enhancement in this subregion might be due to mild microvascular damage. Additionally, the HR features appeared nearly homogeneously distributed across subregions, with similar weighting values, supporting the notion that LNM may originate simultaneously from different regions of the primary tumor. This aligns with Röcken et al’s findings on the evolutionary biology of gastric adenocarcinomas [34], although further validation is needed due to the limited sample size in their study.

There are currently no standardized diagnostic criteria for LNM in tumors. A recent meta-analysis by Zhong J et al identified that the single-score discriminatory ability and generalizability of Node-RADS have not been widely validated and are still in the early stages of clinical application [35]. Therefore, in this study, Node-RADS was not used as a uniform criterion for diagnosing LNM in AEG; instead, the two groups of radiologists of varied seniority were randomly stratified into experience and RADS groups. We observed that for the RADS group, the accuracy of LN diagnosis was notably lower when radiologists assigned Node-RADS scores of 1 or 2 compared to scores of 4 or 5. This resulted in higher false-negative rates for Node-RADS 1 and 2, contributing to lower overall sensitivity compared to specificity in the RADS group. The consistently higher diagnostic accuracy of the HR model across different Node-RADS scores suggests its potential to reclassify false-negative LN with Node-RADS ≤ 2. Similarly, in the empirical group, all four radiologists demonstrated lower sensitivity than specificity in diagnosing LNM in AEG, but HR model assistance clearly improved sensitivity without compromising specificity. Additionally, Loch Florian N et al reported that in CT-based Node-RADS assessments for LNM in gastric cancer, specificity was 90.7% while sensitivity was only 56.8% at the maximum Youden index [28]. In summary, radiologists face a sensitivity deficit in diagnosing LNM in AEG using CT, while the HR model’s high sensitivity aptly fills this diagnostic gap, reducing the risk of missed diagnoses. However, the HR model’s clinical applicability hinges on addressing key challenges such as generalizability, data quality, radiologist acceptance, cost-effectiveness, and seamless integration with existing workflows.

Several limitations exist in this study. First, our patient population was from China, where AEG is predominantly of Siewert types II and III. Therefore, broader external validation of the HR model for Siewert type I is still needed. Second, as this is a retrospective study, it is challenging to correlate tumor tissue samples according to subregion division for HR and pathomics correlation. This could be a prospective research direction for the next step. Lastly, in the routine practice of many imaging departments, diagnostic CT of AEG is made with a portal series alone. Therefore, this study did not take such clinical situations into account.

In conclusion, we have established and validated the ability of the HR model to predict LNM in AEG. This model can serve as a non-invasive, preoperative tool to assist radiologists in better assessing the risk of LNM, especially in patients where radiologists suspect that LN is not metastatic.

## Supplementary information

ELECTRONIC SUPPLEMENTARY MATERIAL

## Data Availability

Data generated or analyzed during the study are available from the corresponding author by request.

## Abbreviations

AEG: Adenocarcinoma of the esophagogastric junction
AUC: Area under the curve
CR: Conventional radiomics
HI: Habitat imaging
HR: Habitat radiomics
ITH: Intra-tumoral heterogeneity
LN: Lymph node
LNM: Lymph node metastasis
Node-RADS: Node Reporting and Data System 1.0
NPV: Negative predictive value
PPV: Positive predictive value
SHAP: Shapley Additive exPlanations
SVM: Support Vector Machine
VEGF: Vascular endothelial growth factor
VOI: Volume of interest
