# Supplementary material for: Habitat radiomics assists radiologists in accurately diagnosing lymph node metastasis of adenocarcinoma of the esophagogastric junction
Source: Insights Imaging. 2025 Apr 24;16:90. doi: 10.1186/s13244-025-01969-9 (PMC12021776; doi:10.1186/s13244-025-01969-9)
Supplement: Supplementary file 1 — ELECTRONIC SUPPLEMENTARY MATERIAL [file 13244_2025_1969_MOESM1_ESM.pdf]

Habitat radiomics assists radiologists in accurately diagnosing lymph node metastasis of adenocarcinoma of the esophagogastric junction

ELECTRONIC SUPPLEMENTARY MATERIAL

Catalogs

Part I Additional description of the experimental procedure

Part II Supplementary tables

Part III supplementary figure

Part I Additional description of the experimental procedure

1.1 Screening of radiomics features

The Z-score algorithm was applied to normalize all radiomics features, ensuring they fell within the same range (mean = 0, variance = 1). Features with an intergroup correlation coefficient (ICC) below 0.75 were excluded, while those with $p < 0.05$, as determined by the t-test, were retained. Redundant features were eliminated using Pearson correlation analysis; if the correlation coefficient between two features exceeded 0.9, one was randomly discarded. Finally, the least absolute shrinkage and selection operator (LASSO) algorithm was employed to select the final feature subset, with the optimal λ value identified through 10-fold cross-validation.

1.2 RADS group of radiologists learning Node-RSDA overview

Node Reporting and Data System 1.0 (Node-RADS), introduced in 2021, is a standardized scoring system designed to categorize lymph node (LN) status in tumors as assessed via CT or MRI scans, following the RADS format, to facilitate more straightforward clinical decision-making [1]. As of now, Node-RADS is primarily utilized in research and has not yet achieved widespread clinical implementation [2]. Consequently, to ensure consistent and reproducible assessment of Node-RADS scores for ln in adenocarcinoma of the esophagogastric junction (AEG) by the two radiologists within the RADS group of this study, a training session on the Node-RADS was conducted. The trainer was G.X., a seasoned radiologist with 15 years of experience in abdominal diagnostics at our institution. The initial training plan spanned 5 days, but due to unforeseen circumstances, it extended to 7 days, with each session lasting 3 hours.

Training Schedule:

Day 1: Introduction to the historical background, significance, and theoretical framework of Node-RADS.

Day 2: Detailed exposition of the specific steps involved in applying Node-RADS, complemented by a practical demonstration using a sample case.

Day 3: Practical exercise involving 20 CT images of AEG along with their clinical data. The two trainees worked through these cases, and upon completion, they were provided with the pathological results to self-correct any errors they might have made.

Day 4: First round of testing using a separate set of 20 AEG CT images and clinical data. The trainees were not informed of the pathological results immediately after completing the test.

Day 11: Second round of testing using the same 20 cases as on Day 4. The consistency of the trainees' results between the two test sessions was evaluated using Fleiss' kappa statistic (κ). At the end of this session, they were informed of the pathological results and any errors they had made, allowing them to self-correct.

Day 18: A second round of repeat testing was conducted using an additional 20 cases, following the same methodology and time interval as before. Fleiss' kappa values were again calculated for both trainees.

The training was designed to continue in this manner until both trainees achieved Fleiss' kappa values greater than 0.8, indicating a high level of agreement and consistency in their Node-RADS scores. All AEG cases used for the radiomics model in the main text.

1.3 Node-RADS scoring process

Node-RADS is a scoring system based on a three-level flowchart that characterizes and scores LN on CT scans to classify them according to their size and configuration. For each subcategory, a score is assigned, and a total score is given to each LN on a scale of 1 to 5, where:

1: Very low
2: Low
3: Indeterminate
4: High
5: Very high

Two primary imaging manifestations are evaluated: size and configuration.

Size:

- Lymph nodes are considered enlarged if their short axis exceeds 10 mm [3].

Configuration:

- Texture: This can be homogeneous, heterogeneous, focal, or macroscopic necrosis.
- Borders: Defined as smooth or irregular.
- Morphology: Can be bean-shaped with fat planes or spherical without fat planes.

These two features—size and configuration—contribute to the final classification of the lymph node.

References

1. Elsholtz Fabian HJ, Asbach P, Haas M et al (2021) Introducing the Node Reporting and Data System 1.0 (Node-RADS): a concept for standardized assessment of lymph nodes in cancer. Eur Radiol 31:6116–6124

2. Zhong J, Mao S, Chen H et al (2024) Node-RADS: a systematic review and meta-analysis of diagnostic performance, category-wise malignancy rates, and inter-observer reliability. Eur Radiol. DOI: 10.1007/s00330-024-11160-1

3. Loch Florian N, Beyer K, Kreis Martin E et al (2023) Diagnostic performance of Node Reporting and Data System (Node-RADS) for regional lymph node staging of gastric cancer by CT. Eur Radiol 34:3183–3193

Part II Supplementary tables

Table S1: CT scanning parameters among multiple centers

Centers	Institution Ⅰ		Institution Ⅱ	Institution Ⅲ		
CT Scanner	SIEMENS Definition Flash	SIEMENS SOMATOM Force	Aquilion 64 TSX-101A	GE Revolution Massachusetts	Aquilion one 320	Aquilion RXL 16
Tube voltage	120kV	120kV	120kV	120kV	120kV	120kV
Tube current	Automatic	Automatic	300mA	Automatic	Automatic	300mA
Gantry rotation time	0.6s	0.5s	0.6s	0.5 s	0.5 s	0.6s
Detector collimation	64×0.625 mm	96×0.625 mm	64×0.5 mm	256×0.625 mm	320×0.5 mm	16×0.5 mm
section thickness	1-5mm	1-5mm	1.25mm	1.25-5mm	1.25-5mm	1.25-5mm
section interval	1-5mm	1-5mm	1.25mm	1.25-5mm	1.25-5mm	1.25-5mm
Pitch	0.8	0.8	0.8	0.8	0.8	1.0
Matrix	512 × 512	512 × 512	512 × 512	512 × 512	512 × 512	512 × 512
Contrast agent concentration	350 mgI/mL	350 mgI/mL	320 mgI/mL	370 mgI/mL	370 mgI/mL	370 mgI/mL
Contrast agent dosage	1.2-1.5mL/kg	1.2-1.5mL/kg	1.0-1.5mL/kg	1.0-1.5mL/kg	1.0-1.5mL/kg	1.0-1.5mL/kg
Contrast agent infused rate	3.0-4.0mL/s	3.0-4.0mL/s	2.5-3.5mL/s	2.5-3.5mL/s	2.5-3.5mL/s	2.5-3.5mL/s
Arterial phase scan	threshold trigger	threshold trigger	threshold trigger	Threshold trigger	threshold trigger	threshold trigger

Institution Ⅰ, Heping Hospital; Institution Ⅱ, Heji Hospital; Institution Ⅲ, China-Japan Friendship Hospital

Table S2 CH value of the K-means algorithm cluster

Number of clusters	Calinski-Harabasz Score
2	2134952.363
3	3075425.408
4	2265194.602
5	1889665.868
6	1742521.084
7	1608679.037
8	1525682.565
9	1453938.625
10	1388785.015

Table S3: Number and class of features extracted by the habitat radiomics (HR) model

Features	Number（n = 546）
shape	n = 0#
grey level co-occurrence matrix (GLCM)	n = 22*3*2
first-order statistics	n = 18*3*2
grey level size zone matrix (GLSZM)	n = 16*3*2
neighborhood gray-tone difference matrix (NGTDM)	n = 5*3*2
grey level run length matrix (GLRLM)	n = 16*3*2
gray level dependence matrix (GLDM)	n = 14*3*2

#, The shapes of the subregions within the habitat model were derived through unsupervised clustering delineation, and since these shapes did not correspond to the original tumor morphology, feature extraction was not conducted for the shapes of the individual subregions.

Table S4: Number and class of features extracted by the conventional radiomics (CR) model

Features	Number（n = 210）
shape	n = 14*2
grey level co-occurrence matrix (GLCM)	n = 22*2
first-order statistics	n = 18*2
grey level size zone matrix (GLSZM)	n = 16*2
neighborhood gray-tone difference matrix (NGTDM)	n = 5*2
grey level run length matrix (GLRLM)	n = 16*2
gray level dependence matrix (GLDM)	n = 14*2

Table S5: Number and class of features extracted by the combined model

Features	Number（n = 756）
shape	n = 14*2
grey level co-occurrence matrix (GLCM)	n = 22*3*2*2
first-order statistics	n = 18*6*2*2
grey level size zone matrix (GLSZM)	n = 16*3*2*2
neighborhood gray-tone difference matrix (NGTDM)	n = 5*3*2*2
grey level run length matrix (GLRLM)	n = 16*3*2*2
gray level dependence matrix (GLDM)	n = 14*3*2*2

Table S6 The prediction performance of six machine learning algorithms in the HR model

Algorithm	Cohort	AUC(95%CI)	Accuracy	Sensitivity	Specificity	PPV	NPV
SVM	Training	0.876(0.8250-0.9279)	0.832	0.902	0.733	0.827	0.840
	Validation	0.869(0.7703-0.9672)	0.808	0.781	0.850	0.893	0.708
	Test	0.795(0.6733-0.9161)	0.779	0.778	0.783	0.894	0.600
Random Forest	Training	0.987(0.9772-0.9969)	0.938	0.943	0.930	0.950	0.920
	Validation	0.723(0.5725-0.8727)	0.689	0.659	0.765	0.879	0.464
	Test	0.783(0.6581-0.9071)	0.818	0.870	0.696	0.870	0.696
ExtraTrees	Training	0.842(0.7876-0.8971)	0.774	0.861	0.651	0.778	0.767
	Validation	0.727(0.5705-0.8841)	0.721	0.705	0.765	0.886	0.500
	Test	0.790(0.6587-0.9210)	0.831	0.889	0.696	0.873	0.727
XGBoost	Training	0.878(0.8327-0.9242)	0.798	0.779	0.826	0.864	0.724
	Validation	0.715(0.5646-0.8659)	0.721	0.795	0.529	0.814	0.500
	Test	0.780(0.6383-0.9221)	0.818	0.870	0.696	0.870	0.696
LightGBM	Training	0.884(0.8404-0.9273)	0.779	0.680	0.919	0.922	0.669
	Validation	0.744(0.5994-0.8886)	0.672	0.614	0.824	0.900	0.452
	Test	0.762(0.6341-0.8893)	0.805	0.889	0.609	0.842	0.700
LR	Training	0.828(0.7710-0.8856)	0.779	0.861	0.663	0.784	0.770
	Validation	0.758(0.6083-0.9077)	0.738	0.750	0.706	0.868	0.522
	Test	0.726(0.5947-0.8578)	0.766	0.815	0.652	0.846	0.600

SVM, Support Vector Machine; RF, Random Forest; ExtRaTrees, Extremely Randomized Trees; XGBoost, eXtreme Gradient Boosting; LightGBM, Light Gradient Boosting Machine; LR, Logistic Regression; PPV, positive predictive value; NPV, negative predictive value; AUC, area under the curve; CI, confidence interval

Table S7 The prediction performance of six machine learning algorithms in the CR model

Algorithm	Cohort	AUC(95%CI)	Accuracy	Sensitivity	Specificity	PPV	NPV
SVM	Training	0.831(0.7718-0.8911)	0.798	0.902	0.651	0.786	0.824
	Validation	0.727(0.5818-0.8713)	0.673	0.687	0.650	0.759	0.565
	Test	0.692(0.5520-0.8328)	0.714	0.796	0.522	0.796	0.522
Random Forest	Training	0.871(0.8224-0.9195)	0.865	0.893	0.826	0.879	0.845
	Validation	0.725(0.5939-0.8561)	0.750	0.844	0.600	0.771	0.706
	Test	0.529(0.3866-0.6708)	0.574	0.636	0.412	0.737	0.304
ExtraTrees	Training	0.984(0.9724-0.9958)	0.933	0.951	0.907	0.935	0.929
	Validation	0.698(0.5418-0.8535)	0.712	0.719	0.700	0.793	0.609
	Test	0.649(0.4785-0.8196)	0.721	0.818	0.471	0.800	0.500
XGBoost	Training	0.830(0.7790-0.8803)	0.615	0.344	1.000	1.000	0.518
	Validation	0.704(0.5439-0.8639)	0.469	0.700	0.714	0.452	0.714
	Test	0.568(0.4158-0.7192)	0.639	0.750	0.353	0.750	0.353
LightGBM	Training	0.755(0.6939-0.8158)	0.393	0.393	0.930	0.889	0.519
	Validation	0.716(0.5798-0.8515)	0.519	0.281	0.900	0.818	0.439
	Test	0.635(0.4868-0.7832)	0.426	0.250	0.882	0.846	0.312
LR	Training	0.809(0.7498-0.8679)	0.750	0.828	0.640	0.765	0.724
	Validation	0.875(0.7736-0.9764)	0.827	0.844	0.800	0.871	0.762
	Test	0.810(0.6750-0.9453)	0.836	0.909	0.647	0.870	0.733

Table S8 The prediction performance of six machine learning algorithms in the combined model

Algorithm	Cohort	AUC(95%CI)	Accuracy	Sensitivity	Specificity	PPV	NPV
SVM	Training	0.865(0.8081-0.9221)	0.841	0.885	0.779	0.850	0.827
	Validation	0.842(0.7296-0.9548)	0.788	0.750	0.850	0.889	0.680
	Test	0.785(0.6578-0.9122)	0.792	0.815	0.739	0.880	0.630
Random Forest	Training	0.987(0.9772-0.9969)	0.938	0.943	0.930	0.950	0.920
	Validation	0.815(0.6805-0.9492)	0.750	0.750	0.750	0.828	0.652
	Test	0.723(0.5725-0.8728)	0.689	0.659	0.765	0.879	0.464
ExtraTrees	Training	0.842(0.7876-0.8971)	0.774	0.861	0.651	0.778	0.767
	Validation	0.811(0.6855-0.9363)	0.788	0.719	0.900	0.920	0.667
	Test	0.727(0.5705-0.8841)	0.721	0.705	0.765	0.886	0.500
XGBoost	Training	0.878(0.8327-0.9242)	0.798	0.779	0.826	0.864	0.724
	Validation	0.812(0.6951-0.9284)	0.673	0.687	0.650	0.759	0.565
	Test	0.715(0.5646-0.8659)	0.721	0.795	0.529	0.814	0.500
LightGBM	Training	0.884(0.8404-0.9273)	0.779	0.680	0.919	0.922	0.669
	Validation	0.831(0.7097-0.9528)	0.750	0.844	0.600	0.771	0.706
	Test	0.744(0.5994-0.8886)	0.672	0.614	0.824	0.900	0.452
LR	Training	0.828(0.7706-0.8853)	0.779	0.861	0.663	0.784	0.770
	Validation	0.806(0.7636-0.8783)	0.746	0.806	0.750	0.853	0.733
	Test	0.758(0.6083-0.9077)	0.738	0.750	0.706	0.868	0.522

Table S9 Hosmer-Lemeshow test for SVM model

Cohort	HR model	CR model	Combined model
Training cohort	0.257	0.537	0.356
Validation cohort	0.274	0.129	0.860
Test cohort	0.246	0.194	0.554

Table S10 Intra-reader consistency analysis of radiologists' results from 3 repeated readings

	Validation cohort				Text cohort			
Readers	Without HM		With HM		Without HM		With HM	
	κ	*p*	κ	*p*	κ	*p*	κ	*p*
Junior 1	0.764	< 0.001	0.821	< 0.001	0.791	< 0.001	0.772	< 0.001
Junior 2	0.782	< 0.001	0.787	< 0.001	0.740	< 0.001	0.701	< 0.001
Junior 3	0.872	< 0.001	0.846	< 0.001	0.878	< 0.001	0.844	< 0.001
Senior 1	0.923	< 0.001	0.870	< 0.001	0.893	< 0.001	0.861	< 0.001
Senior 2	0.858	< 0.001	0.790	< 0.001	0.858	< 0.001	0.827	< 0.001
Senior 3	0.896	< 0.001	0.897	< 0.001	0.890	< 0.001	0.861	< 0.001

κ, Fleiss' kappa statistic

Table S11 Independent diagnosis by radiologists in the RADS Group and HR model predictions in Node-RADS

Node-RADS	Diagnostic index	Radiologist independent diagnosis					HR model predictions				
		Validation		Test		Average value	Validation		Test		Average value
		Junior 3	Senior 3	Junior 3	Senior 3		Junior 3	Senior 3	Junior 3	Senior 3	
1	Accuracy	0.667	0.615	0.476	0.440	0.550	0.889	0.769	0.857	0.840	0.839
	Sensitivity	0.000	0.000	0.000	0.000	0.000	0.667	0.600	0.727	0.786	0.695
	Specificity	1.000	1.000	1.000	1.000	1.000	1.000	0.875	1.000	0.909	0.946
	PPV	0.000	0.000	0.000	0.000	0.000	1.000	0.750	1.000	0.917	0.917
	NPV	0.667	0.615	0.476	0.440	0.550	0.857	0.778	0.769	0.769	0.793
2	Accuracy	0.500	0.500	0.333	0.348	0.420	0.667	0.688	0.762	0.739	0.714
	Sensitivity	0.000	0.000	0.000	0.000	0.000	0.556	0.500	0.786	0.667	0.627
	Specificity	1.000	1.000	1.000	1.000	1.000	0.778	0.875	0.714	0.875	0.811
	PPV	0.000	0.000	0.000	0.000	0.000	0.714	0.800	0.846	0.909	0.817
	NPV	0.500	0.500	0.333	0.348	0.420	0.636	0.636	0.625	0.583	0.620
3	Accuracy	0.733	0.769	0.783	0.824	0.777	0.933	0.923	0.739	0.765	0.840
	Sensitivity	1.000	1.000	1.000	1.000	1.000	1.000	1.000	0.778	0.857	0.909
	Specificity	0.000	0.000	0.000	0.000	0.000	0.750	0.667	0.600	0.333	0.586
	PPV	0.733	0.769	0.783	0.824	0.777	0.917	0.909	0.875	0.857	0.890
	NPV	0.000	0.000	0.000	0.000	0.000	1.000	1.000	0.429	0.333	0.691
4	Accuracy	0.833	0.833	0.857	0.857	0.845	0.833	1.000	0.714	0.714	0.816
	Sensitivity	1.000	1.000	1.000	1.000	1.000	0.800	1.000	0.833	0.833	0.867
	Specificity	0.000	0.000	0.000	0.000	0.000	1.000	1.000	0.000	0.000	0.500
	PPV	0.833	0.833	0.857	0.857	0.845	1.000	1.000	0.833	0.833	0.917
	NPV	0.000	0.000	0.000	0.000	0.000	0.500	1.000	0.000	0.000	0.375
5	Accuracy	1.000	1.000	1.000	1.000	1.000	0.750	0.750	0.800	0.800	0.775
	Sensitivity	1.000	1.000	1.000	1.000	1.000	0.7500	0.750	0.800	0.800	0.775
	Specificity	0.000	0.000	0.000	0.000	0.000	0.000	0.000	0.000	0.000	0.000
	PPV	1.000	1.000	1.000	1.000	1.000	1.000	1.000	1.000	1.000	1.000
	NPV	0.000	0.000	0.000	0.000	0.000	0.000	0.000	0.000	0.000	0.000

Part III supplementary figure

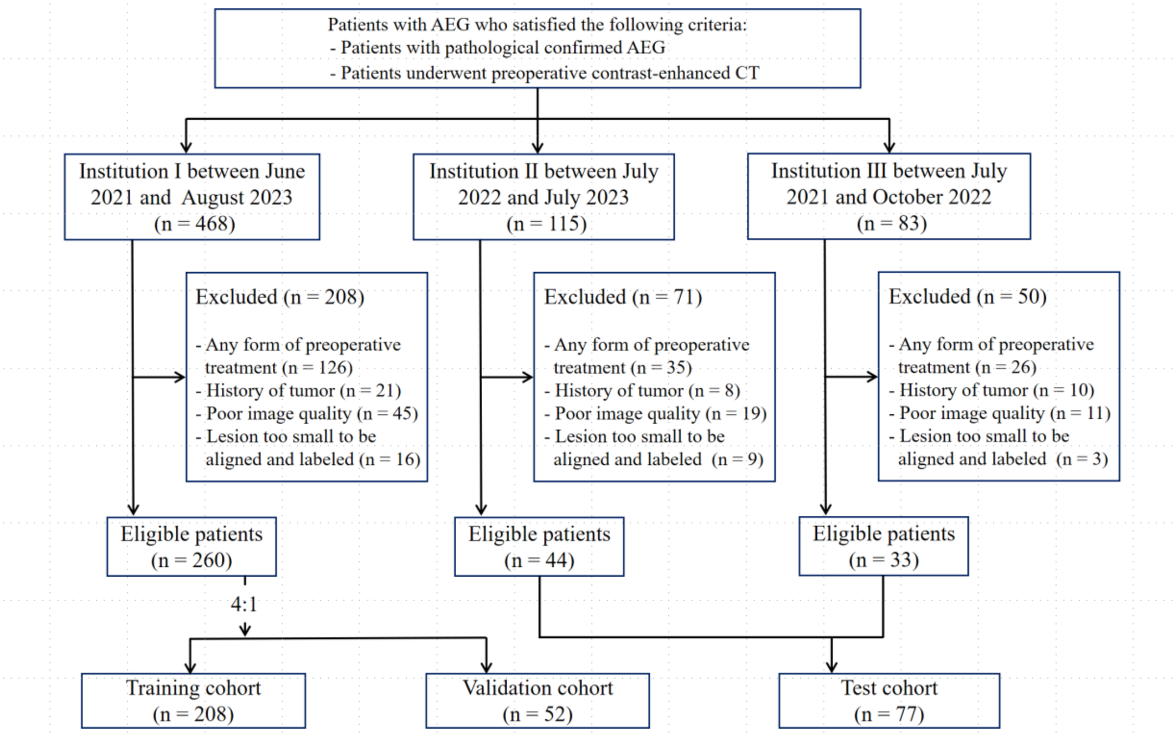

Fig. S1 Inclusion and exclusion criteria flowchart. Institution Ⅰ, Heping Hospital; Institution Ⅱ, Heji Hospital; Institution Ⅲ, China-Japan Friendship Hospital

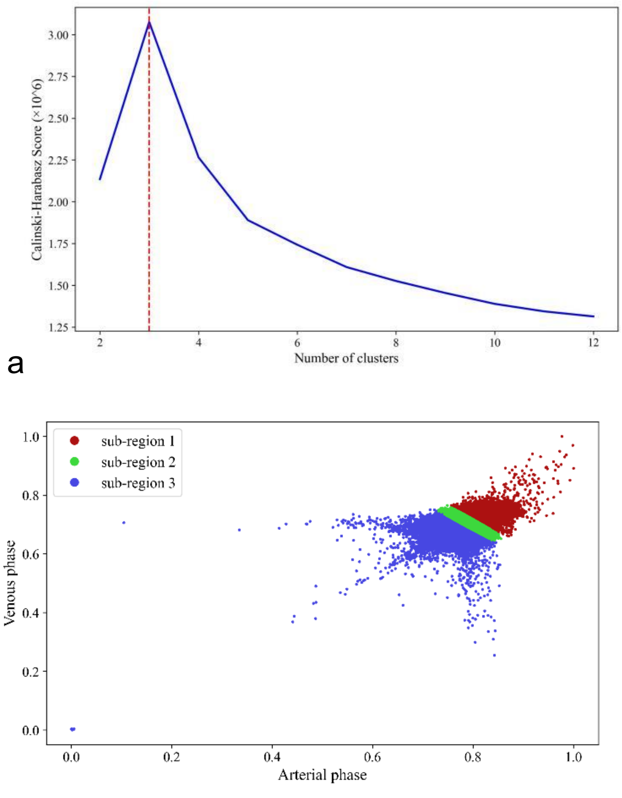

Fig. S2 Habitat K-means clustering results. **(a)** Line graph of Calinski-Harabasz scores. **(b)** Scatterplot of subregional clustering divisions

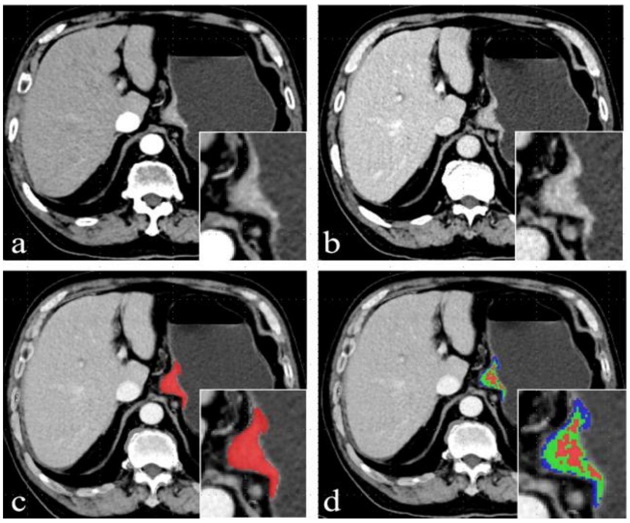

Fig. S3 A case of tumor VOI labeling and subregion delineation in a 56-year-old male patient with AEG (ulcerative type), with local magnification in the lower right corner. **(a, b)** Arterial-phase and venous-phase CT images after alignment, respectively. **(c)** Manual labeling of tumor area. **(d)** K-means clustering divides the tumor into three subregions: red indicates subregion 1, green indicates subregion 2, and blue indicates subregion 3.

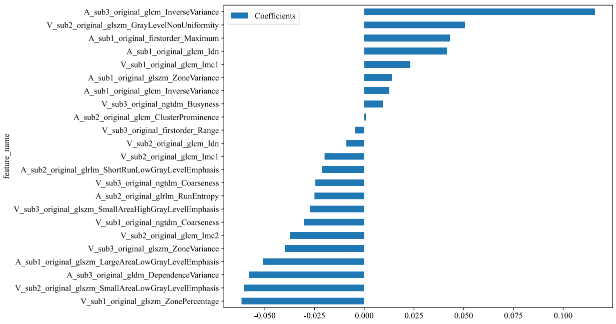

a

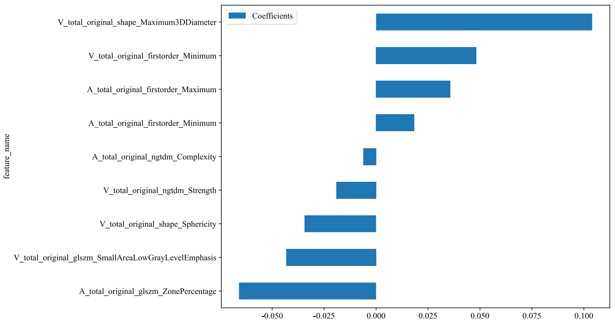

b

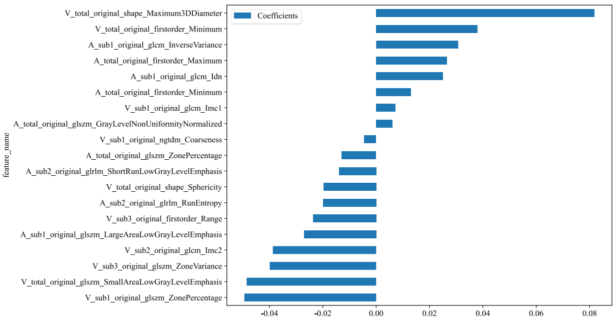

c

Fig. S4 (a) Features of habitat radiomics (HR) screening plot. **(b)** Features of conventional radiomics (CR) screening plot. **(c)** Features of combined radiomics screening plot

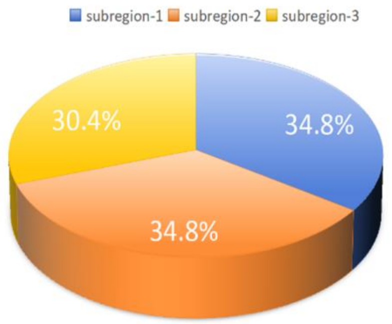

a

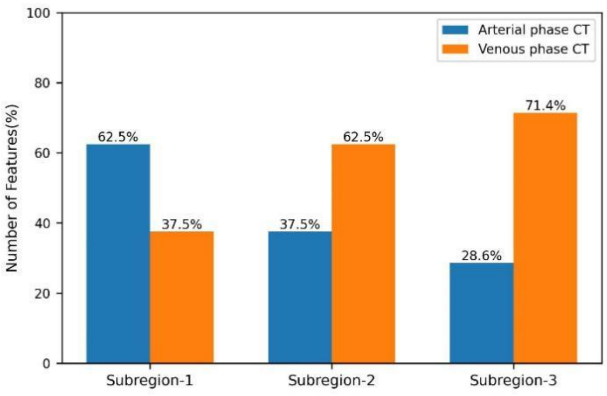

b

Fig. S5 (a) Fan map of subregional distribution of HR features. **(b)** Histogram of phase distribution of HR features

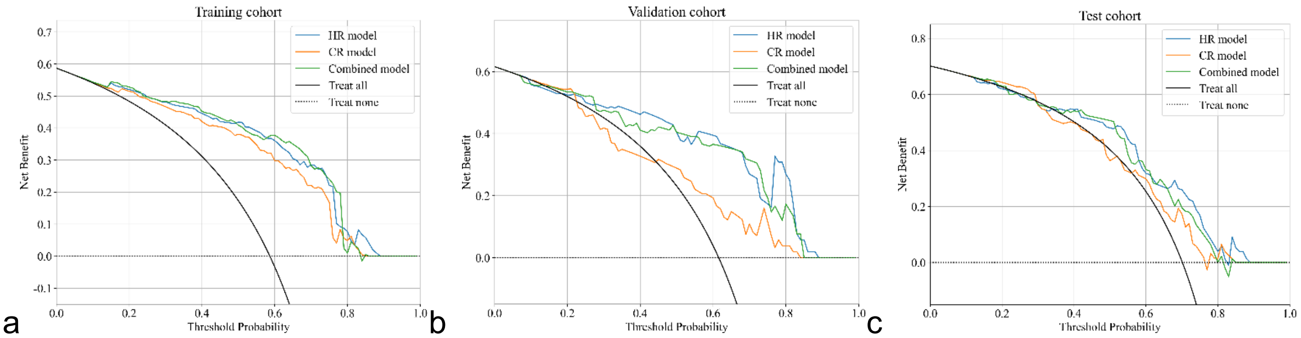

Fig. S6 Decision curve analysis (DCA). **(a)** In the training cohort. **(b)** In the validation cohort. **(c)** In the test cohort

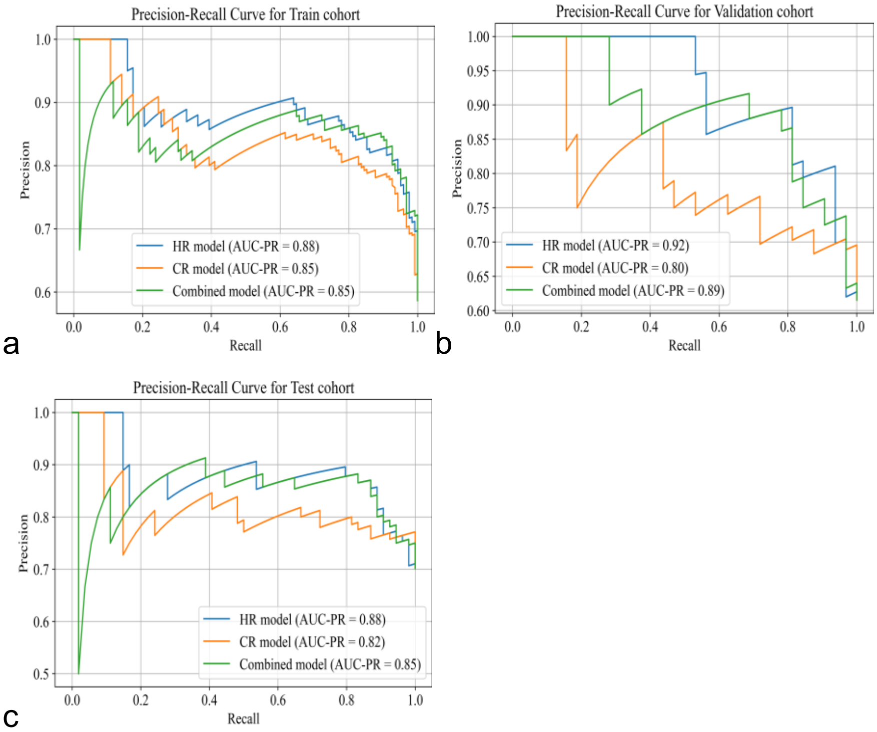

Fig. S7 Precision-Recall curve. **(a)** In the training cohort. **(b)** In the validation cohort. **(c)** In the test cohort

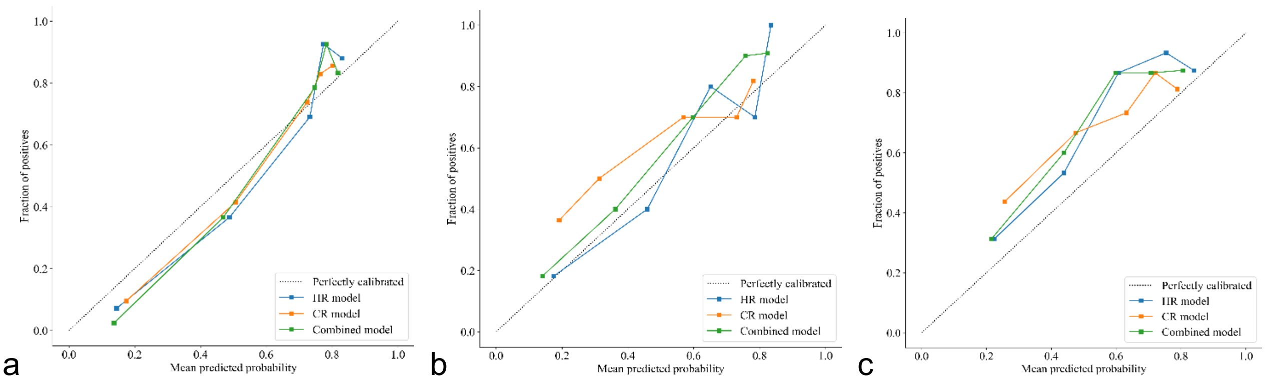

Fig. S8 Calibration curve analysis. **(a)** In the training cohort. **(b)** In the validation cohort. **(c)** In the test cohort

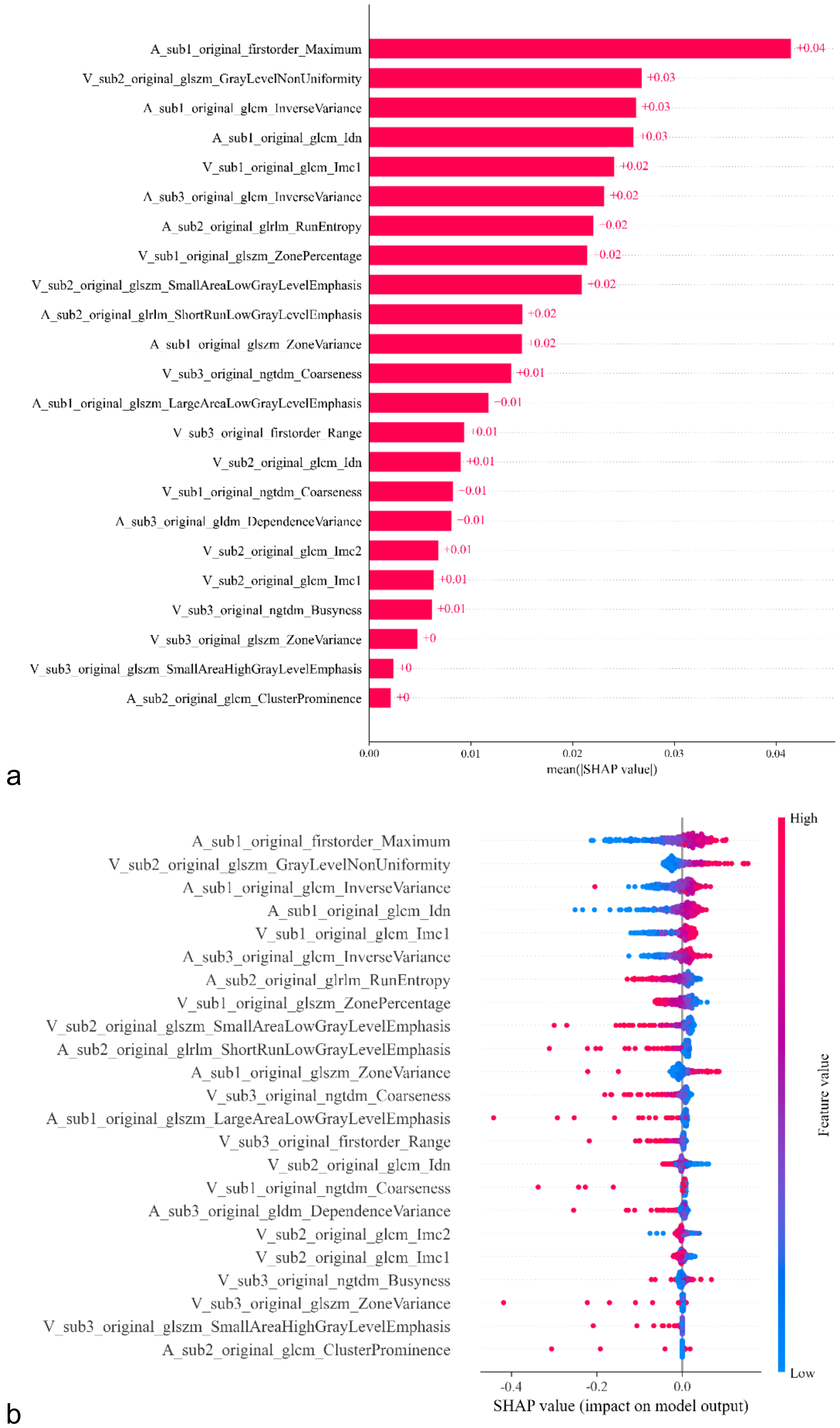
A_sub1_original_firstorder_Maximum +0.04
V_sub2_original_glszm_GrayLevelNonUniformity +0.03
A_sub1_original_glcm_InverseVariance +0.03
A_sub1_original_glcm_Idn +0.03
V_sub1_original_glcm_Imc1 +0.02
A_sub3_original_glcm_InverseVariance +0.02
A_sub2_original_glrlm_RunEntropy +0.02
V_sub1_original_glszm_ZonePercentage +0.02
V_sub2_original_glszm_SmallAreaLowGrayLevelEmphasis +0.02
A_sub2_original_glrlm_ShortRunLowGrayLevelEmphasis +0.02
A_sub1_original_glszm_ZoneVariance +0.02
V_sub3_original_ngtdm_Coarseness +0.01
A_sub1_original_glszm_LargeAreaLowGrayLevelEmphasis +0.01
V_sub3_original_firstorder_Range +0.01
V_sub2_original_glcm_Idn +0.01
V_sub1_original_ngtdm_Coarseness +0.01
A_sub3_original_gldm_DependenceVariance +0.01
V_sub2_original_glcm_Imc2 +0.01
V_sub2_original_glcm_Imc1 +0.01
V_sub3_original_ngtdm_Busyness +0.01
V_sub3_original_glszm_ZoneVariance +0
V_sub3_original_glszm_SmallAreaHighGrayLevelEmphasis +0
A_sub2_original_glcm_ClusterProminence +0
0.00
0.01
0.02
0.03
0.04
mean(|SHAP value|)
a
High
Feature value
Low
-0.4
-0.2
0.0
SHAP value (impact on model output)
b

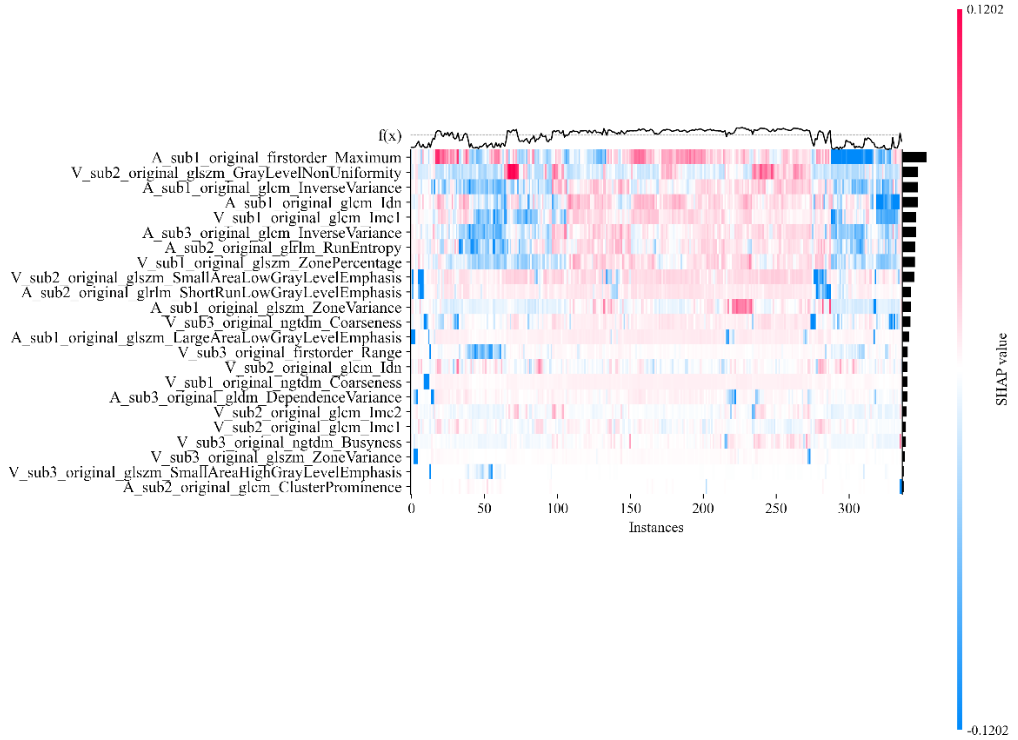

c

Fig. S9 SHAP visualization of HR models**. (a)** Full SHAP bar chart. **(b)** Full SHAP summary plot. **(c)** Full SHAP heatmap

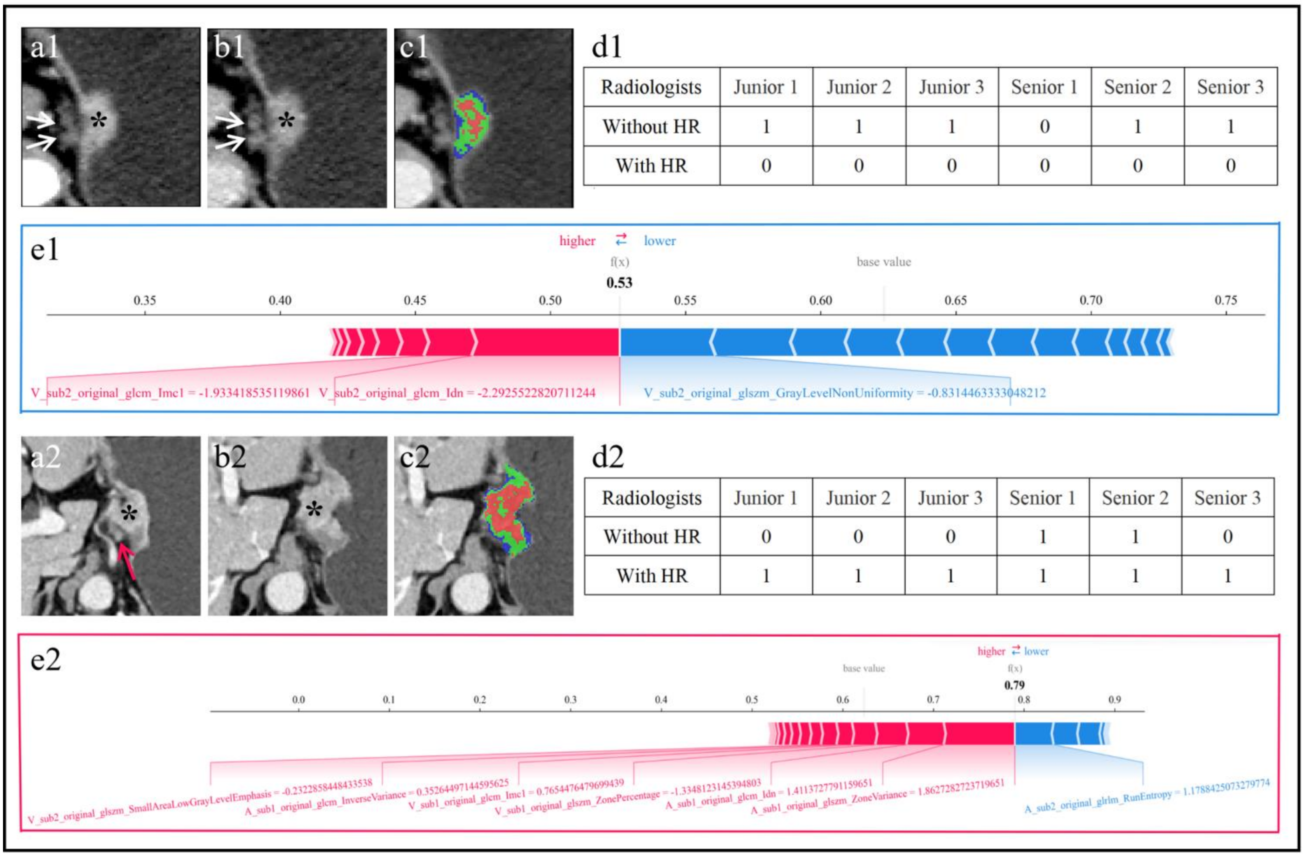

Fig. S10 Individual-Level HR model SHAP force maps and the diagnoses by radiologists with and without model assistance. Patient 1: **(a1-c1)** show two enlarged lymph nodes (LN) (white arrows) on the side of the lesser curvature of the stomach, the larger with a short diameter of approximately 10 mm, which 83% of radiologists considered metastatic at independent diagnosis **(d1)**, with postoperative pathology confirming reactive hyperplasia of the LN, and the force maps HR model predicting the LN to be metastasis-negative **(e1)**, and a reduced diagnostic false-positive rate for radiologists after combining the predictive results of this model. Patient 2: **(a2-c2)** shows a LN (red arrow) with a short diameter of approximately 6 mm lateral to the lesser curvature of the stomach, which was considered non-metastatic by 67% of radiologists at independent diagnosis **(d2)**, and was pathologically confirmed to be metastatic by postoperative pathology, which forcefully demonstrated that the HR Model predicted the LN to be metastasis-positive **(e2)**, and radiologists have a reduced rate of false-negative diagnoses after combining the predictions of this model.
